# The Evaluation of a SEER-Based Nomogram in Predicting the Survival of Patients Treated with Neoadjuvant Therapy Followed by Esophagectomy

**DOI:** 10.3389/fsurg.2022.853093

**Published:** 2022-06-29

**Authors:** Qing Wang, Zhiyong Sun, Xin Xu, Xiumei Ma, Xiaojing Zhao, Qing Ye

**Affiliations:** ^1^ Department of Thoracic Surgery, Renji Hospital, Shanghai Jiao Tong University School of Medicine, Shanghai China; ^2^ Department of Radiation Oncology, School of Medicine, Renji Hospital, Shanghai Jiao Tong University, Shanghai, China

**Keywords:** esophageal carcinoma, esophagectomy, SEER, nomogram, neoadjuvant therapy

## Abstract

**Background:**

A novel nomogram based on the Surveillance, Epidemiology, and End Results (SEER) database has been developed to predict the survival of patients with esophageal carcinoma who received neoadjuvant therapy followed by surgery. We aimed to evaluate the accuracy and value of the nomogram with an external validation cohort.

**Methods:**

A total of 2,224 patients in SEER database were divided into the training cohort (*n* = 1556) and the internal validation cohort (*n* = 668), while 77 patients in our institute were enrolled in the external validation cohort. A Cox proportional hazards regression model was used to develop a nomogram based on the training cohort, while the C-indexes, the calibration curves, receiver operating characteristics curve (ROC), and Kaplan-Meier survival curve were applied in the internal and external validation cohort.

**Results:**

Five independent risk factors were identified and integrated into the nomogram (C-index = 0.645, 95%CI 0.627–0.663). The nomogram exhibited good prognostic value in the internal validation cohort (C-index = 0.648 95%CI 0.622–0.674). However, the C-index, calibration plot, receiver operating characteristics curve (ROC) analysis, Kaplan-Meier survival curve of the nomogram in the external validation cohort were not as good as the training and internal validation cohort (C-index = 0.584 95%CI 0.445–0.723). Further analysis demonstrated that the resection margin involvement (R0, R1, or R2 resection) was an independent risk factor for the patients, which was not included in the SEER cohort.

**Conclusions:**

the nomogram based on the SEER database fails to accurately predict the prognosis of the patients in the external validation cohort, which can be caused by the absence of essential information from the SEER database.

## Introduction

As the eighth most common type of malignant tumor and the sixth leading cause of cancer deaths (1), esophageal carcinoma has caused an estimated 544,076 deaths, with 604,100 new cases worldwide alone in 2020 (2). Despite the continuous efforts to improve the treatment efficacy, many patients are confronted with rapid progression and poor prognosis (3). Many patients presented locally advanced esophageal carcinoma when first diagnosed. Neoadjuvant chemoradiotherapy(nCRT) followed by esophagectomyis recommended as the standard treatment for locally advanced esophageal carcinoma (4), which means T2 to T4a, N0 to N+, and M0 disease, according to the eighth edition of American Joint Committee on Cancer (AJCC) staging system (5).

An accurate and feasible prediction model helps the physicians estimate disease progression and survival of the patients and provide better evidence for clinical practice. Nomogram is a widely used tool for cancer prognosis due to its ability to transfer statistical predictive models into a feasible numerical estimate method (6). Some nomograms have been developed to predict the prognosis of patients with esophageal carcinoma. In 2016, Shapiro and his colleagues developed a nomogram predicting overall survival (OS) exclusively in patients with esophageal carcinoma treated with nCRT and surgery, mainly based on the data derived from the CROSS trial (7). This nomogram contains three factors, including clinical nodal category (cN), pathologic tumor category (ypT), and the number of positive lymph nodes in the resection specimen (ypN). Another study validated the nomogram with 975 patients in three academic centers, demonstrating that the nomogram could accurately predict the OS and progression-free survival (PFS) after nCRT and surgery, with a C-statistic of 0.61 (8).

The log odds of positive lymph nodes (LODDS), defined as the natural logarithm of the ratio of a metastatic lymph node to a non-metastatic lymph node, has been emerging as an essential prognostic factor for cancer prognosis, including colon cancer (9), breast cancer (10), oral squamous cell (11), lung squamous cell carcinoma (12), and so on. For esophageal carcinoma, LODDS also exhibits better discrimination power in risk stratification than N descriptor and positive lymph node ratio (LNR) (13). Ye and his colleagues also developed a nomogram based on the Surveillance, Epidemiology, and End Results (SEER) database, which integrated age, gender, histological grade, T stage, and LODDS as the risk factor, with a C-index of 0.647 (13). However, there was no previous validation by an external cohort, especially in Chinese patients. In this study, we renewed the nomogram by extending the diagnosis year from 2004 to 2016. We validated it using an external cohort of 77 patients with esophageal carcinoma to evaluate the accuracy and value of this nomogram.

## Methods

### Patient Selection

**Figure 1:**
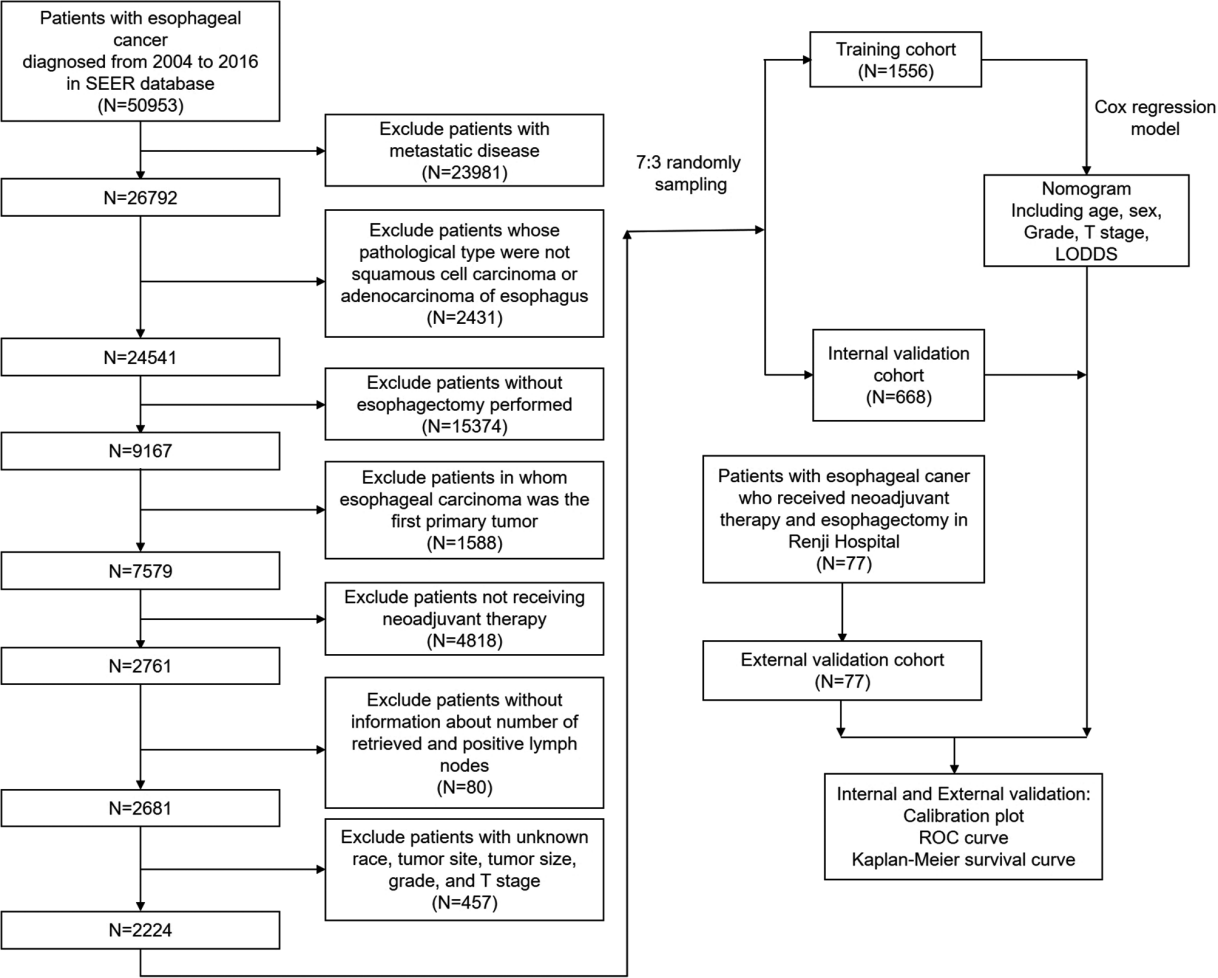
Flowchart of patients screening and study design.

The SEER cohort was selected from the SEER database (http://seer.cancer.gov/). Eighteen population-based cancers were selected in the SEER database, while the SEER*Stat program (v 8.3.9) was used to extract the information of patients with esophageal carcinoma. The extraction conditions were as follows: “the location of the disease: esophagus” and “diagnosis year: 2004–2016.” In the research, we enrolled patients with esophageal carcinoma who received neoadjuvant therapy and esophagectomy between 2004 and 2016. Supplementary Table S1 showed the detailed selection process, Supplementary Table S2 showed the program selection codes, while Figure 1 showed the flowchart of the study design. Following variables were extracted: “Age recode with < 1-year-olds”, “Race recode (White, Black, Other)”, “Sex”, “Year of diagnosis”, “Derived AJCC T, 6th ed (2004–2015)”, “Derived AJCC M, 6th ed (2004–2015)”, “Primary Site - labeled Histologic Type ICD-O-3”, “RX Summ–Surg Prim Site (1998+)”,“CS tumor size (2004–2015)”, “CS Tumor Size/Ext Eval (2004–2015)”, “Grade (thru 2017)”, “Survival months”, “Vital status recode (study cut-off used)”, “Regional nodes positive (1988+)”, “Regional nodes examined (1988+)”, “First malignant primary indicator”. The exclusion criteria were as follows: (a) patients with metastatic disease; (b) patients whose pathological type were not squamous cell carcinoma or adenocarcinoma of esophagus; (c) patients without esophagectomy performed; (d) patients in whom esophageal carcinoma was the first primary tumor; (f) patients not receiving neoadjuvant therapy; (g) patients without information about the number of retrieved and positive lymph nodes; (h) patients with unknown race, tumor site, tumor size, grade, and T stage.

The external cohort was selected from patients with resectable locally advanced esophageal or gastroesophageal junction carcinoma (cT1-T2N+ or cT3-4aNany) who received neoadjuvant chemoradiotherapy followed by esophagectomy from 2015 to 2020 in Renji Hospital. Clinical and pathological data were retrieved retrospectively from the hospital database, while follow-up information was collected by telephone interview. Exclusion criteria: 1. Patients without follow-up data and other essential clinical data; 2. Patients who died of postoperative complications during hospitalization; 3. Patients with surgical resection not completed or time interval between nCRT and surgery more than four months.

### Ethical Statement

The study protocol was approved by the Ethics Committee of Renji Hospital, Shanghai Jiao Tong University School of Medicine (Shanghai, China). Informed consent was obtained from each patient in the external validation cohort, and all personal information was anonymous in the dataset.

### LODDS Calculation

LODDS was calculated by the formula of ln([pLN + 0.5]/[nLN + 0.5]), when pLN meant the number of positive lymph nodes and nLN meant the number of negative lymph nodes. pLN of SEER cohort were variable of “Regional nodes positive (1988+)”, while nLN of SEER cohort were variable of “Regional nodes examined (1988+)”. X-Tile software (version 3.6.1) was used to produce the optimal cut-off of LODDS with the minimal P-value approach. LODDS was divided into three ranges, LODDS1 ≤ −2.8, −2.8<LODDS2 ≤ −1.6, LODDS3 > −1.6.

### Nomogram Development

According to the previous report (14), univariate and multivariate Cox proportional hazards regression models were applied to calculate the hazard ratios (HRs) and corresponding 95% confidence intervals (CIs) of the risk factors for the overall survival of the training cohort. The independent risk factors of the multivariate Cox proportional hazards regression analysis were integrated into the nomogram model. The probability of 1-year, 3-year, and 5-year OS rates could be estimated according to the nomogram.

### Nomogram Validation

The nomogram's discriminative ability and calibration were validated in the training, internal, and external validation cohorts. We used Harrell's C-statistic, or C-index, as the major indicator of the discriminative ability. The C-index values range from 0.5 to 1, meaning the discriminative ability range from none to full. We also used the time-dependent receiver operating characteristic (ROC) curves and the corresponding areas under curves (AUCs) at 1, 3, and 5 years to estimate the discriminative ability. Calibration plots were used for the calibration of the nomogram. The calibration plot is a diagram presenting the relationship between the predicted probabilities and the observed outcomes. The standard curve is a straight line passing through the origin of the coordinate axis with a slope of 1. The more the prediction line falls on a 45-degree diagonal line, the better the model was calibrated (8).

### Statistical Analysis

R software (version 4.0.2) was used to construct the nomogram. A *P* value of less than 0.05 was considered statistically significant. Categorical variables were presented as proportions. Chi-square tests or Fisher's precision probability test were performed in different evaluations of categorical variables. According to the previous report, the sum score of each patient of the three cohorts was calculated based on the Cox hazards proportional regression model. The “surv_cutpoint” function of the “survminer” of the R packages were used to confirm the cut-off point for the risk stratification, which divided the patients into the low-risk and high-risk groups. A Kaplan–Meier survival curve and the log-rank test evaluated the low-risk and high-risk groups (14).

## Results

### Demographical and Clinicopathological Characteristics

Table 1 compared the training and internal validation cohort's demographical and clinicopathological characteristics. A total of 2,224 patients in the SEER database were enrolled in this study and divided into the training cohort and the internal validation cohort with the ratio of 7:3 bootstrapping method. There was no difference between the training and internal validation cohorts in age, race, sex, tumor site, T stage, N stage, histology, histology grade, LODDS, and tumor size (all *P* > 0.05).

**Table 1:** Demographics and clinicopathological characteristics of the training and internal validation cohort.

	Training cohort (*n* = 1556)	Internal validation cohort (*n* = 668)	*P*-value
Age			0.911
20–54 years	338 (21.7%)	142 (21.3%)	
55–64 years	606 (38.9%)	261 (39.1%)	
65–74 years	505 (32.5%)	212 (31.7%)	
75+ years	107 (6.9%)	53 (7.9%)	
Race			0.393
Black	71 (4.6%)	29 (4.3%)	
White	1,423 (91.5%)	613 (91.8%)	
Other	62 (4.0%)	26 (3.9%)	
Sex			0.336
Female	241 (15.5%)	100 (15.0%)	
Male	1,315 (84.5%)	568 (85.0%)	
Tumor site			0.398
Cervical	2 (0.1%)	0 (0%)	
Upper third	14 (0.9%)	12 (1.8%)	
Middle third	170 (10.9%)	83 (12.4%)	
Lower third	1,315 (84.5%)	538 (80.5%)	
Abdominal esophagus	15 (1.0%)	8 (1.2%)	
Overlapping	40 (2.6%)	27 (4.0%)	
T stage			0.794
T1	175 (11.2%)	78 (11.7%)	
T2	261 (16.8%)	131 (19.6%)	
T3	1,034 (66.5%)	410 (61.4%)	
T4	86 (5.5%)	49 (7.3%)	
N stage			0.307
N0	1,025 (65.9%)	439 (65.7%)	
N1	363 (23.3%)	169 (25.3%)	
N2	97 (6.2%)	40 (6.0%)	
N3	71 (4.6%)	20 (3.0%)	
Histology Grade			0.771
Grade I	81 (5.2%)	39 (5.8%)	
Grade II	685 (44.0%)	291 (43.6%)	
Grade III	776 (49.9%)	329 (49.3%)	
Grade IV	14 (0.9%)	9 (1.3%)	
LODDS			0.199
>−1.6	280 (18.0%)	133 (19.9%)	
≤−1.6	536 (34.4%)	216 (32.3%)	
≤−2.8	740 (47.6%)	319 (47.8%)	
Histology			0.554
Adenocarcinoma	1,261 (81.0%)	544 (81.4%)	
Squamous cell carcinoma	295 (19.0%)	124 (18.6%)	
Tumor size			0.735
0–3 cm	384 (24.7%)	179 (26.8%)	
3–5 cm	430 (27.6%)	168 (25.1%)	
5–7 cm	236 (15.2%)	103 (15.4%)	
>7 cm	169 (10.9%)	75 (11.2%)	
Unknown	337 (21.7%)	143 (21.4%)	

However, there were significant differences between the external validation and SEER cohorts in the demographical and clinicopathological characteristics, as shown in Table 2. A total of 77 patients were enrolled in the external validation cohort, and all patients received neoadjuvant radiation therapy. The dose of the radiation before the surgery was 37.8–41.4 Gy per time (average 20 times). In the external validation cohort, patients were younger with no 75+ years (*P* = 0.0285). The external validation cohort was all Chinese patients. The tumor site, T stage, N stage, histology type, histology grade, and tumor size all demonstrated a significant difference between the external validation cohort and the SEER cohort (all *P* < 0.001).

**Table 2:** Demographics and clinicopathological characteristics of the SEER and external validation cohort.

	SEER cohort (*n* = 2224)	External validation cohort (*n* = 77)	*P*-value
Age			0.0285
20–54 years	480 (21.6%)	12 (15.6%)	
55–64 years	867 (39.0%)	33 (42.9%)	
65–74 years	717 (32.2%)	32 (41.6%)	
75+ years	160 (7.2%)	0 (0%)	
Race			<0.001
Black	100 (4.5%)	0 (0%)	
White	2,036 (91.5%)	0 (0%)	
Other	88 (4.0%)	77 (100%)	
Sex			0.18
Female	341 (15.3%)	7 (9.1%)	
Male	1,883 (84.7%)	70 (90.9%)	
Tumor site			<0.001
Cervical	2 (0.1%)	0 (0%)	
Upper third	26 (1.2%)	7 (9.1%)	
Middle third	253 (11.4%)	33 (42.9%)	
Lower third	1,853 (83.3%)	37 (48.1%)	
Abdominal esophagus	23 (1.0%)	0 (0%)	
Overlapping	67 (3.0%)	0 (0%)	
T stage			<0.001
T1	253 (11.4%)	1 (1.3%)	
T2	392 (17.6%)	3 (3.9%)	
T3	1,444 (64.9%)	61 (79.2%)	
T4	135 (6.1%)	12 (15.6%)	
N stage			<0.001
N0	1,464 (65.8%)	4 (5.2%)	
N1	532 (23.9%)	42 (54.5%)	
N2	137 (6.2%)	30 (39.0%)	
N3	91 (4.1%)	1 (1.3%)	
Histology Grade			<0.001
Grade I	120 (5.4%)	16 (20.8%)	
Grade II	976 (43.9%)	34 (44.2%)	
Grade III	1,105 (49.7%)	23 (29.9%)	
Grade IV	23 (1.0%)	4 (5.2%)	
LODDS			
>−1.6	413 (18.6%)	1 (1.3%)	<0.001
≤−1.6	752 (33.8%)	20 (26.0%)	
≤−2.8	1,059 (47.6%)	56 (72.7%)	
Histology			<0.001
Adenocarcinoma	1,805 (81.2%)	12 (15.6%)	
Squamous cell carcinoma	419 (18.8%)	65 (84.4%)	
Tumor size			<0.001
0–3 cm	563 (25.3%)	29 (37.7%)	
3–5 cm	598 (26.9%)	17 (22.1%)	
5–7 cm	339 (15.2%)	13 (16.9%)	
>7 cm	244 (11.0%)	18 (23.4%)	
Unknown	480 (21.6%)	0 (0%)	

### Univariate and Multivariate Analysis in the Training Cohort

We conducted the univariate and multivariate Cox hazards proportional regression analysis to confirm the independent risk factors for the patients' survival, shown in Table 3. Univariate analysis was conducted for the variables of age, race, sex, tumor site, T stage, N stage, histology, histology grade, tumor size, and LODDS, indicating that age, sex, T stage, N stage, grade, and LODDS were significantly associated with the OS of the patients (*P* < 0.05), which were integrated into the multivariate Cox hazards proportional regression model except for N stage which had collinearity with the LODDS. The multivariate model included five variables: age, sex, T stage, grade, and LODDS.

**Table 3:** Univariate and multivariate Cox regression analysis of each factor's ability for predicting OS in the training cohort.

	Univariate analysis		Multivariate analysis	
	HR (95%CI)	*P* value	HR (95%CI)	*P* value
Age				
20–54 years	Reference	0.003	Reference	
55–64 years	1.144 (0.967–1.353)		1.219 (1.030–1.443)	0.021
65–74 years	1.297 (1.092–1.541)		1.403 (1.179–1.670)	<0.001
75+ years	1.522 (1.174–1.974)		1.804 (1.388–2.345)	<0.001
Race				
Black	Reference	0.263		
White	0.819 (0.543–1.235)			
Other	0.788 (0.598–1.040)			
Sex				
Female	Reference	<0.001	Reference	
Male	1.382 (1.152–1.659)		1.342 (1.117–1.612)	0.002
Tumor site				
Abdominal	Reference	0.238		
Cervical	1.196 (0.270–5.302)			
Upper third	0.904 (0.405–2.018)			
Middle third	0.708 (0.399–1.257)			
Lower third	0.667 (0.386–1.154)			
Overlapping	0.962 (0.502–1.845)			
T stage				
T1	Reference	<0.001	Reference	
T2	1.010 (0.785–1.300)		0.984 (0.763–1.267)	0.899
T3	1.452 (1.179–1.788)		1.311 (1.064–1.617)	0.011
T4	1.536 (1.124–2.101)		1.415 (1.033–1.938)	0.030
N stage				
N0	Reference	<0.001		
N1	1.799 (1.561–2.072)			
N2	2.442 (1.939–3.076)			
N3	3.369 (2.600–4.365)			
Histology				
Adenocarcinoma	Reference	0.575		
Squamous cell carcinoma	0.956 (0.817–1.119)			
Grade				
I + II	Reference	<0.001	Reference	
III + IV	1.308 (1.157–1.479)		1.191 (1.052–1.349)	0.006
Tumor size				
0–3 cm	Reference	0.431		
3–5 cm	0.9905 (0.836–1.174)			
5–7 cm	1.0267 (0.841–1.253)			
>7 cm	1.1583 (0.930–1.442)			
Unknown	0.9274 (0.774–1.111)			
LODDS				
≤−2.8	Reference	<0.001	Reference	
≤−1.6	1.849 (1.606–2.128)		1.880 (1.632–2.165)	<0.001
>−1.6	3.187 (2.711–3.747)		3.164 (2.684–3.729)	<0.001

### Nomogram Development

**Figure 2:**
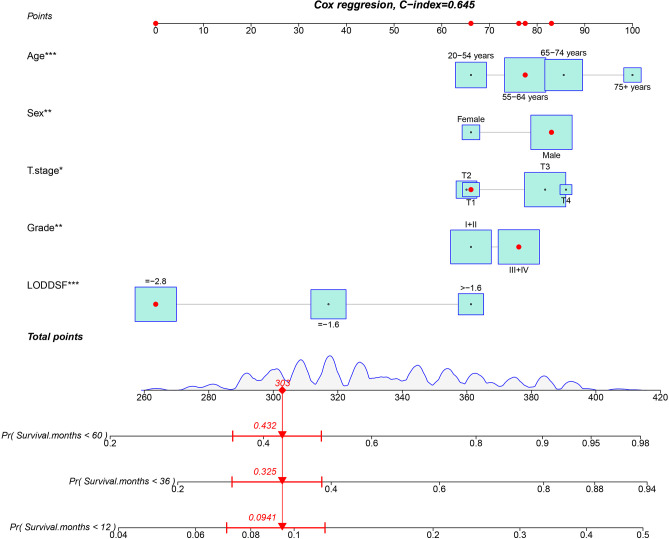
Nomogram for predicting the probability of 1-, 3-, and 5-year overall survival in patients with esophageal carcinoma treated with neoadjuvant therapy.

Based on the multivariate model, we built the nomogram to predict the probability of 1-year, 3-year, and 5-year survival of the patients shown in Figure 2. Age, sex, T stage, grade, and LODDS were independent risk factors. Each variable corresponded to different points. The total point reflects the survival probability by drawing straight down from the total points axis to the 1-, 3-, and 5-year survival axes. For example, a 60-year-old male patient with T1 stage and grade III pathology, as well as LODDS lower than −2.8, he would get a total of 64.1 points, corresponding to the less than 1-, 3-, and 5-year OS probability of 9.4%, 32.5%, and 43.2%, respectively. The C-index of the nomogram in the training cohort was 0.645 (95%CI 0.627–0.663).

### Nomogram Validation

**Figure 3:**
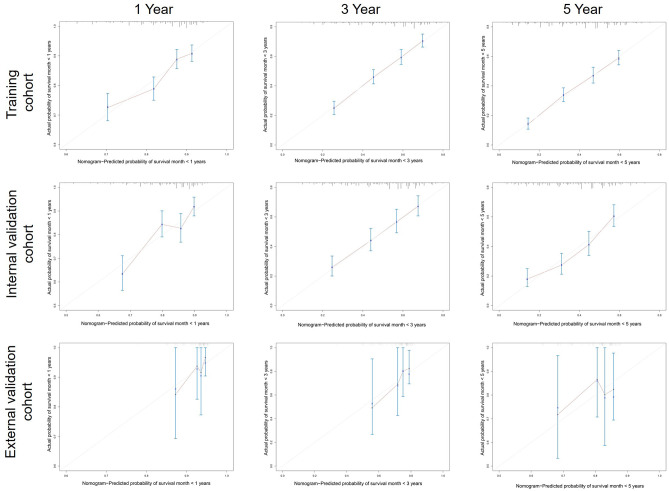
Calibration curves predicting patients’ 1-, 3-, and 5-year OS in the training, internal, and external cohorts.

**Figure 4:**
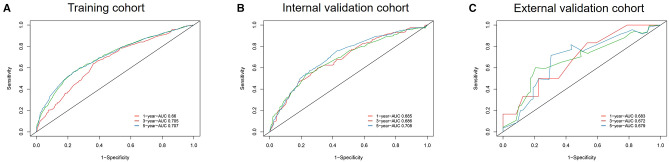
ROC curves and AUCs at 1, 3, and 5 years in the training cohort (**A**), internal validation cohort (**B**), and the external validation cohort (**C**).

A calibration plot was performed to validate the concordance of the nomogram. Figure 3 showed the calibration plot between the nomogram predictions and the actual observed outcomes of the 1-, 3-, and 5-year OS in the training, internal, and external validation cohorts. The calibration plot demonstrated favorable consistency in the training and internal validation cohorts. However, when the nomogram was applied in the external validation cohort, the consistency was not as good as in the training and internal validation cohorts. The C-index of the nomogram in the internal validation cohort was 0.648 (95%CI 0.622–0.674), while the C-index in the external validation cohort was 0.584 (95%CI 0.445–0.723). ROC analysis presented similar results, as shownin Figure 4. The 1-year- AUC of the training, internal, and external validation cohorts were 0.660, 0.685, and 0.683, respectively. The 3-year- AUC of the training, internal, and external validation cohorts were 0.705, 0.686, and 0.672, respectively. The 5-year- AUC of the training, internal, and external validation cohorts were 0.707, 0.708, and 0.679, respectively. The AUCs of the external validation cohort were lower than the training cohort and internal validation cohort.

**Figure 5:**
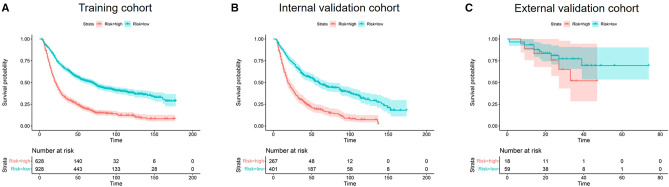
Kaplan–meier curves of OS for risk stratification in the training cohort (**A**), internal validation cohort (**B**), and the external validation cohort (**C**).

The Cox hazard proportional regression model's cut-off point was set at 1.31, shown in Supplementary Figure S1, which divided the patients into low-risk and high-risk groups. The low-risk and high-risk groups exhibited significantly different OS in training and internal validation cohorts (both *P* < 0.001). However, there was no statistical significance between OS of the low-risk group and high-risk group in the external validation group (*P* = 0.3) (Figure 5).

### Cox Regression Analysis in the External Cohort

To explore the potential reasons for the different behavior of the nomogram in the internal and external validation cohort, we conducted a univariate and multivariate Cox hazards proportional regression analysis in the external validation cohort, with more clinical data included, such as postoperative complications and surgical type (R0 resection or not), shown in Supplementary Figure S3 and Table S3. Although the age, sex, T stage, grade, and LODDS were independent risk factors in the SEER cohort (Supplementary Figure S2), those factors didn't show statistical significance in univariate and multivariate analysis. However, we found that the resection margin involvement (R0 resection or not) was a significant risk factor both in the univariate analysis (R1 or R2 resection, HR 7.912, 95%CI 2.199–28.47, *P* = 0.010) and multivariate analysis (R1 or R2 resection, HR 4.957, 95%CI 0.783–31.380, *P* = 0.089). When integrating the age, sex, histology, T stage, LODDS, grade, and resection margin involvement into the model, the concordance of the multivariate Cox hazards proportional regression model was 0.692 (95%CI 0.494–0.746).

## Discussion

Neoadjuvant therapy followed by surgery is currently the treatment of recommendation from major international societies for locally advanced esophageal carcinoma. It is reported that up to 32% of patients show a complete pathological response (ypCR) after neoadjuvant therapy (15). This study developed a novel nomogram that integrated several essential factors including age, sex, T stage, histology grade, and LODDS, based on a cohort including 2,224 patients in the SEER database. This nomogram showed reasonable discrimination and calibration ability in the training cohort and internal validation cohort. However, when it was applied in an external validation cohort of 77 patients in a Chinese thoracic surgery center, the discrimination and calibration became relatively unfavorable. Plenty of nomograms have been developed for patients with esophageal carcinoma in different ways (16), including the adenosquamous esophageal carcinoma (17), early-onset esophageal carcinoma (18), metastatic esophageal carcinoma (19), and so on. Semenkovich and his colleagues developed and validated a nomogram predicting the likelihood of occult lymph node metastases in surgically resectable esophageal carcinomas, including histology, tumor stage, tumor size, grade, and presence of lymphovascular invasion (20). Compared with Semenkovich's study, the histology and tumor size were not significant risk factors in our study's Cox proportional hazard regression analysis. Another study evaluated the prognosis of esophageal carcinoma patients with stages I–III with a nomogram, which consisted of age, marital status, sex, T_stage, N_stage, grade, and surgery (21). The disparity of included factors could be attributed to a different database, targeted patients, and baseline characteristics.

A previous published nomogram established a cohort of 626 patients who underwent nCRT plus surgery, with cN, ypT, and ypN categories included (7). The C-index of the nomogram was moderate at 0·63. Goense and his colleagues used an international multi-institutional cohort of patients to validate this cohort. They found that the discriminative ability of the nomogram for OS was moderate (C-statistic, 0.61) and comparable to that of the initial cohort (C-statistic, 0.63), and the nomogram was also beneficial for the prediction of PFS (C-statistic, 0.64) (8). This nomogram was very simple and easy to use. However, many critical factors were neglected, including the demographical data and histology grade. Ye et al. compared the discriminatory power and value of N descriptor, LNR, and LODDS in the survival prediction of patients with esophageal carcinoma receiving neoadjuvant therapy (13). They found that LODDS demonstrated a higher discriminatory power and goodness of fit over N descriptor and LNR.

Furthermore, they developed a novel nomogram based on a SEER cohort of 2,239 patients, including sex, age, grade, T stage, and LODDS. However, they never validated it in an external cohort. In this study, we updated the SEER cohort with the 2016 added, and rescreened the patients with more strict inclusion criteria. Finally, we enrolled 2,224 patients in the analysis and divided them into the training and internal validation cohorts. We've reached similar results with Ye's study and built a nomogram integrating age, sex, grade, T stage, and LODDS. Age and sex were commonly used in many nomograms when male patients and older patients had worse prognoses (17). Patients with poorly differentiated or undifferentiated histology grades faced a higher risk of recurrence and metastasis and a worse prognosis (22). T stage and LODDS were correlated with the TNM staging system and affected the prognosis. We've noticed no significance between T1 and T2 stages in the multivariate regression analysis, and Ye's study had similar results, which could be attributed to the sample size. Compared with the traditional N descriptor (TNM staging system), LODDS is a novel and promising ratio-based lymph node (LN) staging system reported in many malignant tumors. Yu and his colleagues proved that LODDS exhibited better predictive performance than the N descriptor, the number of positive lymph nodes (NPLN), and LNR among patients with node-positive lung squamous cell carcinoma after surgery (12). However, Baqar et al. reported that LODDS didn't show advantages over LNR and recommended using LNR given its ease of calculation (9). In this study, we adopted the LODDS as the major predictor of the lymph node indicator, which is significant in both univariate and multivariate regression analysis.

The Cox regression model and nomogram showed favorable discrimination and calibration ability in the SEER cohort, with a C-index of 0.645 (95%CI 0.627–0.663) in the training cohort and a C-index of 0.648 (95%CI 0.622–0.674) in the internal validation cohort, which was comparable with the previous study. Nevertheless, the C-index was only 0.584 (95%CI 0.584–0.723) in the external validation cohort. The ROC analysis showed similar results: the 3-year- AUC and 5-year- AUC of the nomogram in the training and internal validation cohort were higher than in the external validation cohort. We used the nomogram to stratify patients' risk in different cohorts and compared their survival curves. The high-risk and low-risk groups showed survival differences without statistical significance in the external validation cohort. The disparity between the external validation and SEER cohorts could be attributed to the following reasons. First, the external validation cohort patients were all Chinese, who were others (American Indian/AK Native, Asian/Pacific Islander) in the SEER cohort. Although the race variable was no significance in the risk factor analysis, the race difference might still affect the prognosis. In the external validation cohort, all patients were Chinese, while the race of Asian was a minority and categorized with other races in the SEER cohort. Second, much important information was missed from the SEER database.

We added the resection margin involvement (R0 or R1, R2 resection) in the multivariate regression analysis and found that the resection margin involvement was an independent risk factor for the prognosis. When added into the model, the C-index of the nomogram reached 0.692 (95%CI 0.494–0.746), significantly higher than the previous result. Third, the neoadjuvant therapy and the surgery plan varied between the SEER cohort and the patients in our hospital, which might affect the final results. Different surgery types (Ivor Lewis and McKeown esophagectomy) could affect the short-term efficacy and prognosis (23, 24). Last, the histology type difference between the SEER cohort and the external validation cohort might be another reason for the inconsistency. Squamous cell carcinoma was the primary histology type in Asians, while the proportions of adenocarcinoma were higher in Western patients (25). Semenkovich et al. developed a nomogram for predicting node-positive disease in esophageal cancer and found that adenocarcinoma was a significant risk factor for node-positive disease compared with squamous (20). On the contrary, Du and his colleagues demonstrated that squamous was a risk factor for cancer-specific survival of patients with esophageal carcinoma after resection. Interestingly, many studies didn't find the significance of histology for the survival of the patients (8, 26), including ours. The effect of histology on the survival of patients with esophageal carcinoma requires further investigation.

Several limitations of this study must be noted. First, the sample size of the external validation cohort was not as significant as in other studies due to study limitations. As a result, the result of the Kaplan-Meier curve in the external validation cohort might be a false-negative mistake. Also, the Cox regression analysis for the external validation cohort was not converged, and many essential risk factors didn't show statistical significance. The sample size of 20 times the number of factors in the nomogram is proper for validation. In our case, a sample size of 100 patients would be better, but a sample size of 77 cases is enough to find important risk factors. Even with the limited sample size, we showed that the resection margin involvement (R0, R1, or R2 resection) was an independent risk factor, indicating that the nomogram was not accurate due to the lack of this variable. Second, the C-index of the nomogram was not perfect and applicable, which requires more studies to incorporate novel prognostic variables. Amulti-institutional cohort might be more potent than a single-center study to improve and validate the nomogram. Last, recent years have witnessed substantial progress in targeted therapy and immunotherapy, shifting the landscape of neoadjuvant therapies for locally-advanced esophageal carcinoma. As a result, genetic mutation status and the administration of novel therapies can greatly affect the prognosis, absent from the SEER database and the nomogram.

## Conclusions

For patients treated with neoadjuvant therapy followed by surgery, the SEER cohort-based nomogram in the external validation cohort was not as descriptive and accurate as in the internal validation cohort. The nomogram failed to predict the prognosis in the external validation cohort of Chinese patients and should be applied with caution. Future studies should incorporate more prognostic variables to improve the nomogram's descriptive ability and application value.

## Data Availability

The original contributions presented in the study are included in the article/Supplementary Material, further inquiries can be directed to the corresponding author/s.
