# Supplementary figures and images for: The Evaluation of a SEER-Based Nomogram in Predicting the Survival of Patients Treated with Neoadjuvant Therapy Followed by Esophagectomy
Source: Front Surg. 2022 Jun 29;9:853093. doi: 10.3389/fsurg.2022.853093 (PMC9276989; doi:10.3389/fsurg.2022.853093)

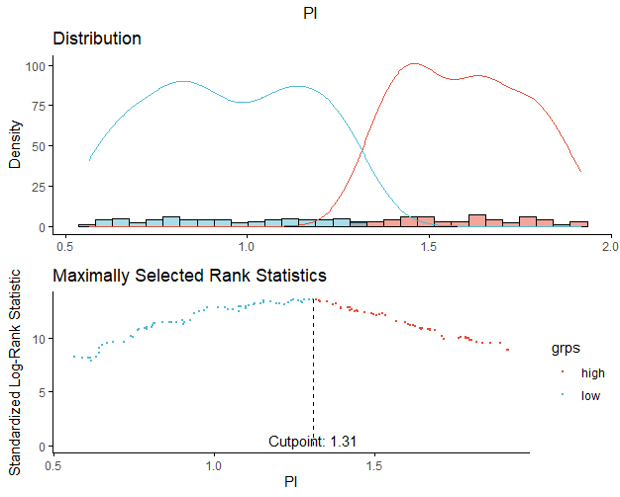

Supplement: Supplementary file 1 [file Image_1_v1.jpeg]

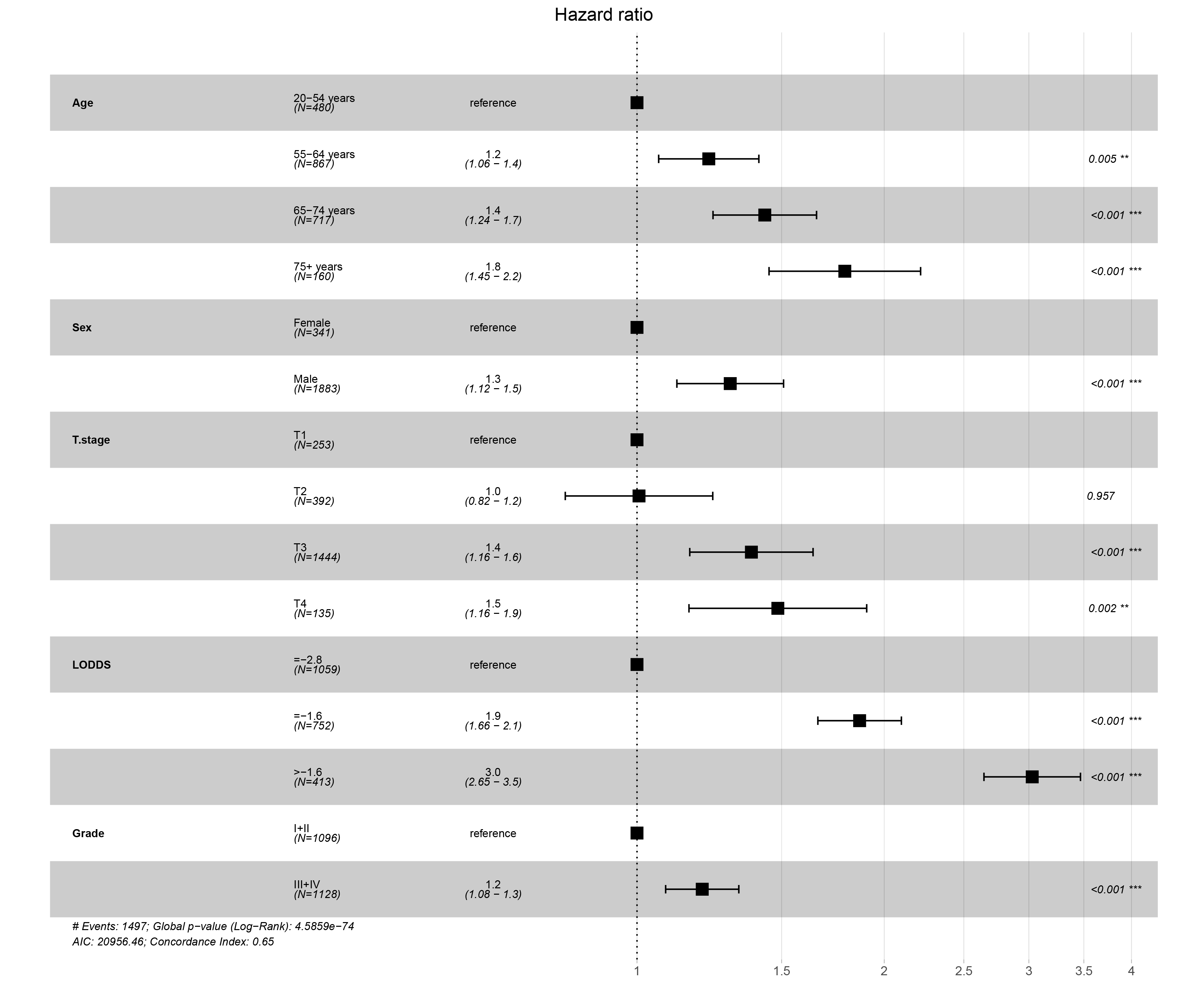

Supplement: Supplementary file 2 [file Image_2_v1.jpeg]

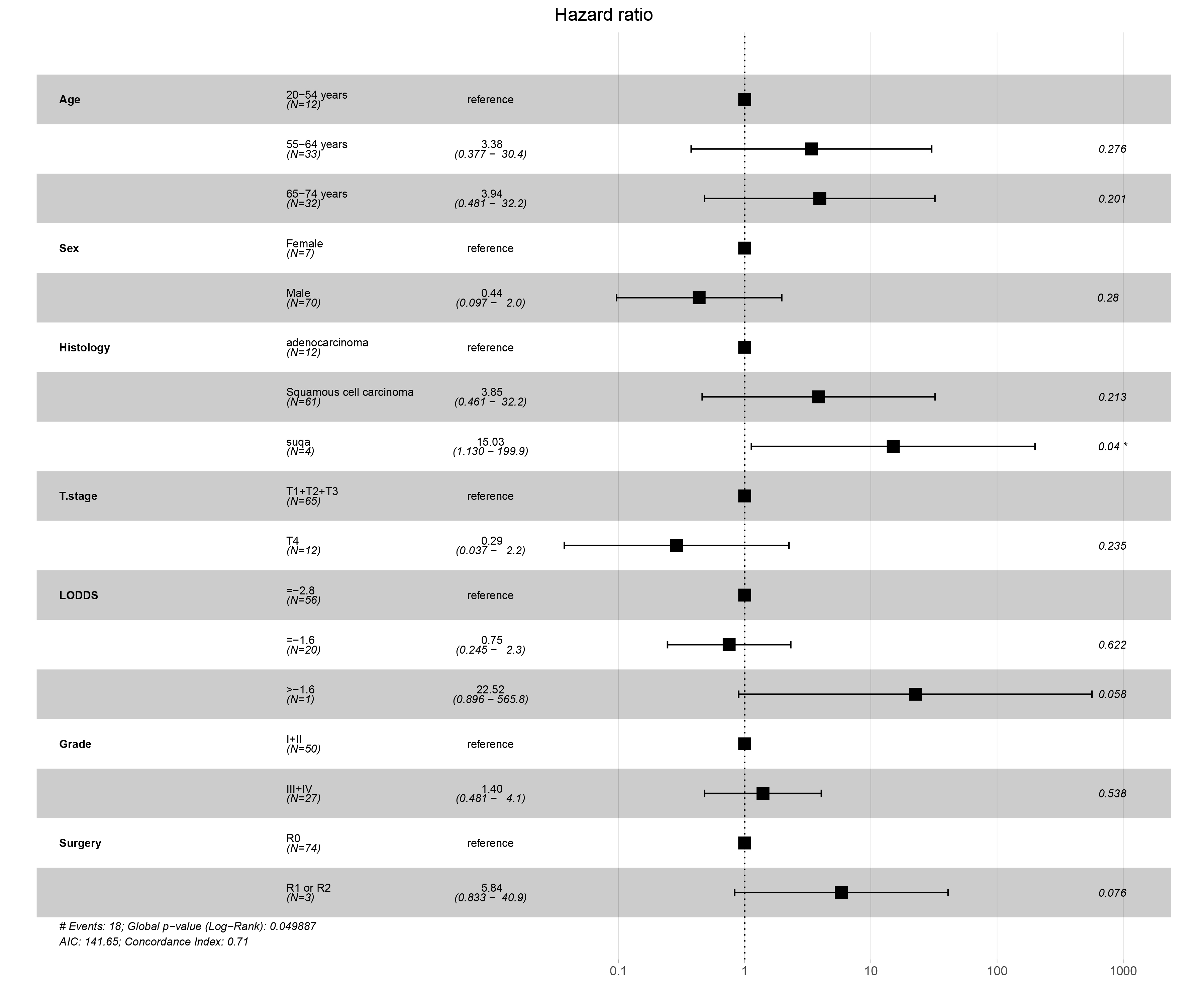

Supplement: Supplementary file 3 [file Image_3_v1.jpeg]
